# A cluster randomized controlled trial to assess the efficacy of a telehealth-based train-the-trainer mealtime intervention delivered by respite care center volunteers to caregivers of persons with dementia to improve nutritional outcomes and quality of life

**DOI:** 10.1186/s40795-020-00350-x

**Published:** 2020-06-24

**Authors:** Elaine J. Amella Krug, Suparna Qanungo, Kelley L. Martin, Martina Mueller, Mohan Madisetti, Teresa J. Kelechi

**Affiliations:** 1 College of Nursing, Medical University of South Carolina, 99 Jonathan Lucas Street, MSC 160, Charleston, SC 29425-1600 USA; 2 Digestive Disease Center, Medical University of South Carolina, 5 Charleston Drive, MSC 198, Charleston, SC 29425 USA; 3 Department of Public Health Sciences, Medical University of South Carolina, 135 Cannon Street, MSC 835, Charleston, SC 29425 USA

**Keywords:** Alzheimer’s disease, Dementia, Weight loss, Caregiver, Respite care, Train-the-trainer, Nutrition

## Abstract

**Background:**

Persons with dementia with mild to moderate cognitive impairment are at risk for developing impairments with activities of daily living such as the ability to feed oneself, that negatively influence health. Lack of caregiver skills related to mealtime planning for persons with dementia and the ability to cope with dysfunctional behaviors are well-documented factors that influence nutritional status outcomes, lead to weight loss, poor quality of life, and impact their ability to remain at home.

**Methods:**

A cluster randomized controlled trial with a parallel mixed methods evaluation processes will be conducted to examine the efficacy of a train-the-trainer program using non-paid volunteers in respite care centers to deliver a telehealth mealtime intervention guided by the theory-based *C3P Model*—*Change the Person, Change the People, Change the Place (C3P).* In this six-month study, dyads of caregivers and persons with mild to moderate dementia receiving respite care services will be randomized to receive either the telehealth intervention or enhanced usual care. Within the intervention group, dyads will be partnered with a C3P trained volunteer who will work with caregivers via videoconference to devise and implement mealtime plans. Under usual care, dyads will receive standardized educational materials modified from *The Savvy Caregiver Program* for Alzheimer’s disease. The primary outcomes include weight maintenance or gain of the person with dementia and quality of life of the caregiver. A multi-level evaluation process utilizing respite center administrators and directors, volunteers, and caregivers will explore intervention fidelity, acceptability and sustainability. Using both the CONSORT and SPIRIT checklists as guidance, the comprehensive study design is more fully described in this manuscript.

**Discussion:**

In this trial, we will lay the groundwork to examine the efficacy and sustainability of a train-the-trainer telehealth program that could be widely disseminated by national Alzheimer’s organizations and readily adopted by community agencies to provide additional resources to assist families in managing mealtimes at home, while promoting the quality of life of both the caregiver and the person with dementia.

**Trial registration:**

This study was registered with clinicaltrials.gov: NCT03622814 on August 9, 2018..

## Background

Alzheimer’s Disease and other related dementias (ADRD) such as Vascular Disease, Lewy Body Disease, and Frontotemporal Disorders that primarily occur among older adults can affect the ability to perform many routine activities of daily living (ADLs). One ADL that affects persons with moderate cognitive decline is the ability to prepare and perform the necessary steps to consume appropriate foods [1]. Using appropriate utensils, consuming healthy food offered in appropriate amounts, preparing food safely, choosing an appropriate diet, and as the disease progresses, recognizing hunger and accepting food offered by others becomes severely compromised [2]. The ability to complete these mealtime tasks is often lost in a predictable progression; the last capacities lost are the ability to recognize what is appropriate to eat, and the ability to feed oneself [3, 4]. Staff in organizations and institutions that serve older persons with dementias (PWD) and their family caregivers need to plan for these changes as eating problems result in weight loss and nutritional deficits that negatively affect health. Prior to institutionalization, eating problems are often seen among older adults who are enrolled in community-based Respite Care Centers (RCC) or Adult Dementia Daycare [5]. In the U.S., it is anticipated that the number of PWD will rise to 14 million over the next four decades [6]. Thus, the community capacity to serve family caregivers will need to increase to accommodate the numbers of PWD served and have the ability to expand the types of resources offered, to allow families to remain together in the home.

Living at home for as long as possible with support is widely promoted as the best living situation for the PWD [7]. Included in that assumption is that the PWD is able to eat enough to sustain him or herself with the supervision or physical help of a family member or caregiver. The four most recent national assessments, the 1999 and 2004 National Long Term Care Survey, and the 2011 and 2015 National Health and Aging Trends Study showed an increase in family caregiving versus institutional (nursing home) care in the U.S. [8].These surveys also showed that in the past decade a significant number of older people who are cognitively impaired live alone and do not have fulltime caregivers who, among coordinating other tasks, are able to prepare, provide and supervise two to three meals daily. Most people eat better in social surroundings; it is no different for PWD [9]. Thus, most programs for older people in both community based and residential care settings involve some element of socialization around meals.

For 60 years, RCC has been a way for people with mild to moderate dementia to remain in the home longer or avoid institutionalization all together [10]. In the U.S, a small trained staff and volunteers (VOLs) provide art, music, and other mentally stimulating programing such as current events discussions over 4 h per day up to 5 days per week. The PWDs attending RCCs must have an accountable caregiver, and all centers are required to offer both a morning snack and lunch meal. Thus, the respite care staff and volunteers become familiar with the wants and needs of their ‘clients’ (PWDs) and are able to adjust both social events and mealtimes to accommodate their client’s individual needs [11]. It is this accommodation that fits within the C3P Model (Change the Person, People and Place) developed by Amella (1999) that has been pilot tested [1] and is currently being tested in this larger study.

Based on the Bronfenbrenner and Evans (2000) Social Ecological Model [12], the C3P Model is a theory based mealtime intervention that teaches an approach to both VOLs at RCCs and caregivers at home that focuses on three intertwined components: What does the Person with Dementia want to eat based on his or her history and preferences such as personal needs, lifelong eating habits, or needs related to medical health history or cultural identity (such as religious beliefs); Who are the People involved in meals for the PWD? (i.e. the family, caregivers, or RCC staff); and, How does the Place where meals occur affect the PWD’s capacity to eat? (e.g. is it a cluttered or an orderly environment). These components form a fundamental background that allow meals to become a social event that many older people still cherish and in which they will eat with more comfort when provided. The underlying premise is that when an older person is encountering problems with meals, caregivers should look beyond just the food itself, to the environmental factors for possible changes that could facilitate eating. Thus eating is a not a simple ADL but one that requires examination of and changes to the context of meals to allow the PWD to be placed in the best situation to eat, have involved and knowledgeable caregivers, and be exposed to a pleasant environment where meals are served in familiar ways.

This study will evaluate the efficacy of a novel intervention Partners at Meals (PaM) that empowers caregivers and volunteers in RCCs who interact with PWD during mealtime. Specifically, we will implement a train-the-trainer program, derived from the theory based C3P Model that will be delivered by telehealth and used to improve PWD nutritional outcomes and quality of life for both PWDs and caregivers. Using a cluster randomized controlled trial design in conjunction with a parallel mixed methods process evaluation, the primary hypothesis of this study is that participation in PaM will be associated with improved PWD body weight or weight maintenance and decreased dysfunctional mealtime behaviors compared to the control group receiving enhanced usual care.

## Methods/design

### Study design

**Fig. 1:**
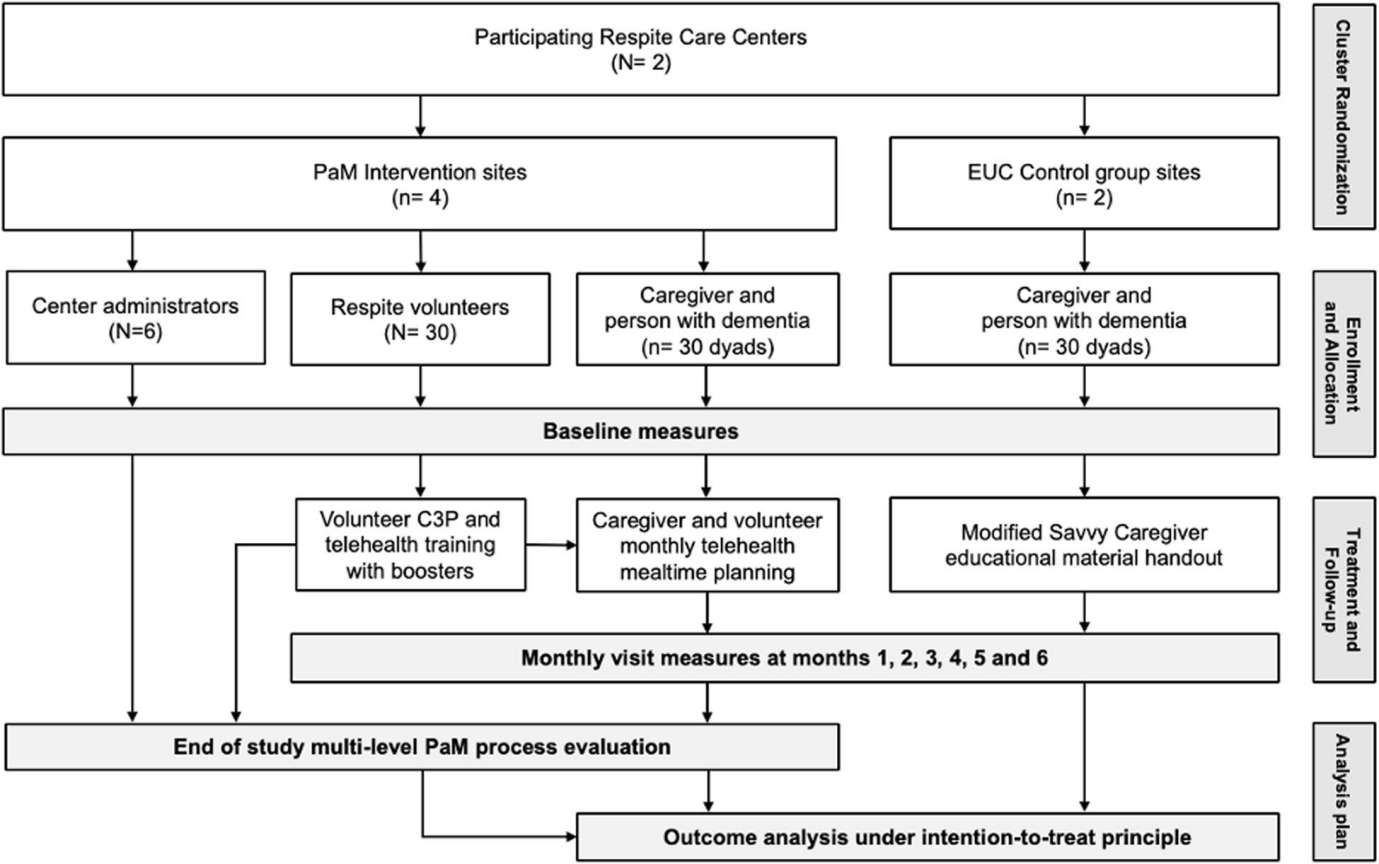
Diagram of the study flow

This is a multi-site, facility-level cluster randomized controlled trial. Participating RCCs will be randomly allocated to the PaM telehealth intervention or the control enhanced usual care group. Volunteers, caregivers, and PWDs will be allocated to each study arm and receive study instruction based upon the site they attend. In-home or on-site study visits will occur monthly over a six-month study period. Volunteers, caregivers, and RCC site administrators and directors will all participate in a mixed methods process evaluation at the end of their study involvement. The overall flow of this study is outlined in Fig. 1.

### Setting and participants

The study will be conducted at 6 individual RCC sites from two large not-for-profit community-based respite care organizations and among a network of home respite care providers in the Southeastern United States.

### Recruitment and eligibility criteria

#### Respite care centers and administrators

Both study site RCC organizations and their executive directors have long-standing relationships with one of the lead investigators (EAK), and both organizations have partnered with the investigator on prior shared funded research projects. Eligibility criteria for administrators included worked with the organization for at least 3 months and planning to be in place for at least 9 more months.

#### Respite center volunteer

At each PaM study site, the researchers will hold study presentations in group settings for non-paid volunteers to gauge study interest. If the volunteer is interested, eligible, and provides written informed consent, he or she will be enrolled into the study. Eligibility criteria include: 18 years of age and older; volunteers at a participating RCC at least once a week for 4 h; must speak English; identify as comfortable in the teacher/coach role; demonstrate ability to use telehealth technology platform; willingness to attend intervention training and follow the 6-month study protocol; and, provide written informed consent. Individuals who receive any payment for their services are excluded.

#### Caregiver and person with dementia

At both PaM and control group study sites, the researchers will work directly with facility administrators and directors to develop and implement a site-specific caregiver recruitment plan. Participant recruitment dissemination strategies will include study advertising through the use of IRB approved flyers and brochure, the direct mailing of letters, and, group presentations to caregivers. Those interested are directed to contact the researchers for more information or for how to join the study. If the caregiver and PWD are both interested, eligible, and provide consent, they are enrolled into the study.

Eligibility criteria for caregivers include: resides with or on the same property as the PWD; provides 4 h or more of care/day; assists with ADLs, including meals; demonstrates ability to use telehealth technology platform; willingness to follow the 6-month study protocol; and, provide written informed consent. Caregivers who received any payment for their services are excluded.

Eligibility criteria for the PWD include: 45 years of age and older; attending a participating RCC at least once per week (intervention or control group) or receiving other weekly RCC services (control group only); living with or within the same property as caregiver; diagnosis of Alzheimer’s disease or related dementia with mild to moderate stage as demonstrated by the Functional Assessment Staging Scale (FAST) of 4 or greater; absence of wasting disorders (i.e., HIV/AIDS, heart or renal failure or COPD, end-stage cancer); some supervision required; mild dysfunctional behavior present (e.g., redirection) at meals; ability to provide verbal assent or written informed consent, or has a legally authorized representative who provides written informed consent by proxy; not receiving enteral feeding; not receiving active treatment by a speech pathologist/therapist; and, not diagnosed with dysphagia as identified by caregiver. Those PWD enrolled in or qualifying for hospice are excluded.

### Randomization and blinding

Based upon RCC facility characteristics (such as the number of clients and their demographics), individual facilities will be grouped to balance size and racial diversity, and then these clusters will be randomly allocated to either the PaM intervention or usual care control group. The allocation sequence will be generated by the study biostatistician using a computer generated model; caregivers and PWDs will be enrolled into each study arm based on their site attendance for respite care. Program implementation issues such as travel to study sites to provide PaM volunteer training prevent the research team from being blinded to site allocation; however, individual caregivers (CG) and PWD allocation in the study database are only accessible to the study coordinator who collects participant data and the members of the study’s Data and Safety Monitoring Committee when reviewing adverse events.

### Sample size and power calculation

Sample size and power calculations were based on two-sided pooled t-test of mean differences between weight changes at the follow-up visit in the two groups, intervention (PAM) and enhanced usual care group. The Type I error rate (alpha level, or significance level) was 0.05 (two-sided). Sample size requirements were adjusted to account for a three-level (RCC, VOL, PWD and CG) cluster randomization scheme, using the variance inflation factor method described by Teerenstra et al. (2008) [13]. Sample size estimate and power calculations were performed for within RCC sample sizes of 6–12 volunteers and 2–4 PWD / caregiver within each volunteer in each group, resulting in 24 PWD / caregiver dyads within each RCC. Intracluster correlations were assumed to range from 0 to 0.1 for ICC_PC_ and 0–0.05 for ICC_V_. Power calculations were based on an effect size (Δ, between group difference) of 0.95 standardized units (units of standard deviation, s). Assuming a drop-out proportion up to 25% of participants, a total of approximately 60 PWD/CG dyads per group are needed.

### Intervention

Based on the C3P model, the PaM intervention involves a train-the-trainer process that includes RCC volunteers who are trained to train caregivers of PWDs in adaptive mealtime strategies and techniques to develop their caregiving skills in the home.

Training is comprised of two initial sessions (each 60–90-min) delivered by a C3P trained research team member who is also trained in adult education. The trainings will occur at the PaM intervention sites and will be repeated as needed for all new site volunteers. These initial sessions include: an introduction to dementia, dysfunctional behaviors, and mealtime issues; mealtime assessments, planning, and adaptive strategies and techniques to promote feeding and nutrition; principles of adult education and teaching skills; and, instructional use of a Health Insurance Portability and Accountability Act (HIPPA) compliant telehealth platform Doxy.me for volunteer/caregiver interaction. Before and after the completion of training, caregivers will complete a pre- and post mealtime knowledge test and self-efficacy survey. Volunteers will be provided with a study manual, that includes all training topic materials for reference, meal planning and assessments forms, and all other required study case record forms such as meal logs and meeting checklist forms.

After receiving their PaM training, volunteers at intervention sites will be matched with a consenting CG/PWD dyad from the same site. The trained volunteer will then interact with the caregiver for a period of six-months with the intent to hold monthly telehealth meetings to review meal plans and continue to train the caregivers on skills around mealtime. At their initial in-person baseline meeting, the C3P trained team member will observe and make additional recommendations/suggestions to the volunteer and caregiver to 1) assess presenting PWD mealtime behaviors using the Edinburgh Feeding in Dementia Scale (EdFED) with additional behavior item (XL); 2) complete a C3P checklist that focuses on changes that could be made in the Place, People or Person; 3) develop a monthly meal plan that incorporates the strategies/suggestions to bring about the change in the 3Ps; and, 4) provide instructional training to the caregiver in the use of the telehealth platform and use of a study tablet to take pictures of meals and record mealtime activity while at home. Both caregivers and volunteers are provided with WIFI ready-enabled tablets and data plans for use while enrolled in the study.

During study months 1–6, each month PWD’s weight and mid-arm circumference will be measured by the research team member and caregivers will be requested to complete a schedule of monthly measures (see Table 1). Each month, caregivers will also be asked to maintain a detailed meal log and use the tablet to take a photo of 3 meals (a breakfast, lunch, and a dinner), and to take a 5–10 min video recording of an everyday meal with the PWD and upload these files to a secure server for viewing by the volunteer and subsequent analysis by the researchers. Additionally, caregivers and volunteers will meet monthly over the Doxy.me telehealth video platform to review and revise meal plans as determined by the PWDs current level of functioning, nutritional intake, and any other health conditions or cultural factors such as religious beliefs. At least 1 monthly meeting will be observed by the research team to assess volunteer skills competency and to monitor fidelity to the C3P program. Volunteers will be provided with booster training sessions (60–90 min) if they exhibit a lack of mastery of their C3P training and also if they are assigned to a dyad more than 2 months after their initial training.

**Table 1:** SPIRIT diagram displaying schedule of enrollment, interventions, and assessment

	ENROLLMENT	ALLOCATION	POST- ALLOCATION						CLOSEOUT
TIMEPOINT	-t_1_	0	Month 1	Month 2	Month 3	Month 4	Month 5	Month 6	t_x_
ENROLLMENT									
Volunteer, PWD, and CG pre-eligibility screening checklist	X								
Informed consent	X								
Eligibility screen	X								
PWD Mini Mental Status Examination (MMSE)	X								
PWD Functional Assessment Staging Scale (FAST)	X								
Allocation (cluster)		X							
INTERVENTIONS									
Volunteer recruitment and training	X								
PaM telehealth program (intervention)			X	X	X	X	X	X	
Enhanced usual care (control)			X	X	X	X	X	X	
ASSESSMENTS									
Person with Dementia (PWD)	X								
Demographics and characteristics	X								
Weight and mid-arm circumference (MAC)	X		X	X	X	X	X	X	
Edinburgh Feeding in Dementia Scale (EdFED) + extra behavioral questions	X		X	X	X	X	X	X	
Quality of Life in Alzheimer’s Disease (QOL-AD)	X		X	X	X	X	X	X	
Caloric intake (analyzed from pre and post photos of meals)			X	X	X	X	X	X	
Dysfunctional behaviors (analyzed from mealtime video recordings)			X	X	X	X	X	X	
Caregiver (CG)									
Demographics and characteristics	X								
Dementia and Mealtime knowledge test (pre/post)	X							X	
C3P Self-efficacy for change (pre/post)	X							X	
Center for Epidemiological Studies – Depression Scale (CES-D)	X		X	X	X	X	X	X	
Zarit 12-item Burden Scale	X		X	X	X	X	X	X	
European Quality of Life (Euro-QL)	X		X	X	X	X	X	X	
Program satisfaction survey								X	
Semi-structured interview								X	
Volunteer									
Demographics and characteristics	X								
Dementia and Mealtime knowledge test (pre/post)	X								
Training satisfaction survey	X								
C3P Self-efficacy for change (pre/post)	X							X	
Telehealth caregiver meeting logs			X	X	X	X	X	X	
Meal plan forms			X	X	X	X	X	X	
Program satisfaction survey								X	
Semi-structured interview								X	
Administrator									
Demographics and characteristics	X								
Program satisfaction survey								X	
Semi-structured interview								X	

At the end of the study, volunteers, caregivers, and RCC site administrators and managers will complete an exit survey and participate in semi-structured interviews with the researchers to assess program satisfaction and to identify multilevel contextual factors (barriers and facilitators) to program implementation, adoption, and sustainability.

### Control

In the control or enhanced usual care group, dyads do not receive the volunteer-facilitated PaM intervention but will receive selected and researcher developed standardized Alzheimer’s caregiving literature.

At the baseline visit, caregivers enrolled in the usual care group will meet with the researchers and will be provided with a WIFI-enabled tablet with a data plan and a study manual that includes: an introduction to the study, study instructions, required study case record forms, basic information on health and nutrition, and selected modified caregiver educational materials from The Savvy Caregiver Program for Alzheimer’s Caregiving [14]. These selected and modified educational materials include basic strategies for dementia caregiving and cover topics such as dealing with emotions, making decisions, elements of caregiving and self-care, communication skills, and challenging issues such as coping with behavior of the PWD and dealing with social isolation. Similar to the PaM intervention group, all caregivers will also complete monthly meal logs and take and upload photos of a breakfast, a lunch and a dinner using the tablet provided to a secure server. They will not capture mealtime video recordings. All other baseline and monthly outcome measures including quality of life, weight, arm circumference, calorie intake, mealtime behaviors, self-efficacy, caregiver burden (Table 1) for the caregivers and PWDs will be completed by the research team. Every attempt will be made to include PWDs with similar levels of cognitive/behavioral disabilities in both the PaM and usual care groups; however, because this is cluster design, equal balance cannot be assured.

### Outcome measures and instruments

As noted in the aims, there are key outcome measures as well as mediating and moderating variables to address secondary aims. The primary measures against which the study is powered lie with the PWD are weight change and change in dysfunctional behavior. The SPIRIT diagram (Table 1) summarizes the study’s schedule of enrollment, interventions, assessments and timing by participant type**.**

### Data collection, management, and retention

At each data collection time point, data are directly entered by the researchers into a Research Electronic Data Capture (REDCap) database hosted on secure servers at the Medical University of South Carolina. Individual participant paper source documents and case record forms are kept securely locked in a filing cabinet and will be maintained for 6 years post-study completion per institutional policy. Only IRB-approved study personnel with the appropriate delegated responsibilities by the principal investigator will have access to the study database and records.

### Statistical methods

#### Outcome analysis

Descriptive statistics will be used to characterize the study sample in terms of demographic and clinical features as appropriate. The intent-to-treat sample will comprise of all participants with at least one post-baseline measurement. In primary analyses, change in outcome measures for the PWD, caregiver and volunteer will be compared between the groups using a generalized linear mixed models (GLMM) approach with PaM/enhanced usual care as the primary independent variable and the primary outcome measures as the dependent variable in individual models [15–17]. Group (PaM/enhanced usual care) will be included as a fixed effect; terms representing the cluster effects RCCs and volunteers (for PWD and caregiver outcomes) will be included in the model as random effects to account for correlation among PWD/caregiver dyads within the same volunteer, as well as for volunteers within the same RCC. In a second step, models will be adjusted for covariables such as age, race/ethnicity, gender, marital status, MMSE, years since diagnosis, location of RCC (rural vs. urban) and other putative prognostic factors. Though standard dyadic analysis of these data is not possible since different instruments are used for assessments of the individual members of the dyad due to the nature of the underlying disease of the PWD, we will adjust models for relevant covariates obtained from the caregiver to account for influences of caregiver burden, self-efficacy, depression and quality of life on PWD outcome measures.

Further, the adjusted average number of hospitalizations/institutionalizations in the PaM and enhanced usual groups will be compared using a GLMM approach as described above. Odds ratios for the categorical outcome measure hospitalized / institutionalized (yes/no) adjusted for covariables will be obtained using logistic regression modeling analogous to the GLMM approach described above. Process evaluation, and feasibility measures including RCC administrator satisfaction will be reported as appropriate. Frequency distributions of adverse events (AE) and serious adverse events (SAE) will be determined for the two groups. All AEs and SAEs will be collected, assessed, and tracked through to final resolution by the researchers per institutional policy. Proportions within categories of adverse events for PaM compared to enhanced usual care usual will be compared via chi-square analyses. All analyses will be conducted using SAS Statistical Software Version 9.4 (Copyright© 2016 by SAS Institute Inc., Cary, NC, USA).

#### Process evaluation

A multi-level mixed-methods process evaluation involving the review of individual case records and developed meal plans as well as one-on-one semi-structured interviews and surveys with RCC administrators, program directors, volunteers and caregivers will be used to examine the contextual factors (barriers and facilitators) on the delivery and efficacy of the PaM telehealth intervention.

The aims of the process evaluation are to:

i. explore contextual factors at all levels related to the delivery of the intervention;
ii. assess volunteer and caregiver change in knowledge related to mealtime planning and coping with dysfunctional behaviors among PWD
iii. assess volunteer and caregiver fidelity to the intervention;
iv. explore participants experiences related to the intervention (reach, acceptability and satisfaction); and,
v. explore the likelihood of program adoption, sustainability, and replicability.

For process evaluation, the distribution of RCC administrator, program director and caregiver satisfaction with the program and willingness to continue the program post-funding will be examined and reported in the intervention group only. Further, the proportion of CG uploading all meal observation recordings (adherence) will reported for adherence, proportions of CGs and volunteers reporting technology problems will be determined as feasibility measures.

In-person semi-structured interviews will be conducted by a single member of the research team and will last approximately 45–60 min. All interviews will be recorded, transcribed and then thematically analyzed. A structured interview guide based on the RE-AIM framework [18] and codebook will be developed and used to assign interviewee responses to one of the four constructs (Reach, Adoption, Implementation, Maintenance). Specific questions including how the program was introduced to caregivers, the barriers and facilitators to implementation, and factors including impacting the program’s sustainability at the RCC will be included. Thematic analysis and interpretation will be performed by three members of the research team until consensus is reached, to increase the scientific rigor and validity of the findings.

### Data and safety monitoring

Participant safety, and the ethical treatment of this vulnerable population are of paramount importance. A Data and Safety Monitoring Committee comprised of an independent dysphagia expert (PhD, CC-SLP), a independent family nurse practitioner (RN, PhD), an independent technology for healthy lifestyle expert (PhD), the study biostatistician (PhD), and the trial director (MS) will convene semi-annually to review all adverse events, monitor the study safety profile, and make recommendations regarding study modification, termination, and continuance.

### Respite care center and participant study compensation

For every year of the study, the two partnering RCC organizations will each receive $10,000 compensation for the needed extra resources, facility space, staff time, and operating costs associated with the conduct of this study at their sites. Trained volunteers will receive $100 for each PWD/caregiver dyad they work with for at least one month; there is no further remuneration. The total number of dyads is 5 with which any volunteer can work; and, thus the maximum compensation for volunteers is $500. All caregivers enrolled in the study, whether in the PaM or usual care group, will receive $50 in compensation at enrollment and $50 per month for every month they are enrolled in the 6-month study for a maximum of $350. It is expected that because the caregivers live with or on the same property as the PWD, that this money will be considered shared.

### Dissemination

We plan to disseminate and share the findings from this study both locally within our community and academically through presentations at relevant conferences for Alzheimer’s Disease researchers, professionals specializing in gerontology, and other healthcare professionals, as well as in peer-reviewed publications.

## Discussion

In the U.S., with an increasing aging population, the need and demand for community based RCCs are also expected to increase as a direct resultant. This cluster RCT will evaluate the efficacy of an innovative train-the-trainer model targeting volunteers at RCCs in the telehealth delivery of a theory based mealtime intervention among caregivers of PWD with mild to moderate cognitive impairments. By focusing on patient-centered outcomes related to dysfunctional mealtime behaviors and through the use of a mixed method and multi-level approach to understand and explore contextual factors to the delivery of this innovative intervention, we lay the groundwork to examine the potential for the sustainability of a program that could be readily disseminated and adopted by national organizations and community agencies alike. The goal is to build capacity and offer resources remotely to families around managing mealtimes for this vulnerable population, so that they may remain at home, where they prefer.

## Data Availability

The data obtained in the current study will be available from the corresponding author upon reasonable request after publication of the results on the main research questions.

## Abbreviations

AE: Adverse event
ADL: Activities of daily living
C3P: Changing the Place, People and Person
CES-D: Center for Epidemiological studies - Depression scale
CG: Caregiver
CONSORT: Consolidated Standards for Reporting of Trials
EDFED XL: Edinburgh feeding in dementia scale + extra dysfunctional behavior questions
EUC: Enhanced Usual Care
EURO-QOL: European quality of life
FAST: Functional Assessment Staging Test
GLMM: Generalized linear mixed models
MAC: Mid arm circumference
MMSE: Mini Mental Status Exam
PaM: Partners at Meals
PWD: Persons with dementia
QOL: Quality of life
QOL-AD: Quality of life in Alzheimer’s Disease
REDCap: Research Electronic Data Capture
RCC: Respite care center
RCT: Randomized controlled trial
SAE: Serious adverse event
SMC: Data and Safety Monitoring Committee
SPIRIT: Standard Protocol Items: Recommendations for Interventional Trials
VOL: Volunteer

## References

[1] Amella EJ, Batchelor-Aselage MB (2014). Facilitating ADLs by caregivers of persons with dementia: the C3P model. Occup Ther Health Care.

[2] Batchelor-Aselage M, Amella EJ, Rose SB, Bales CW (2015). Dementia-related mealtime difficulties: Assessment and management in long-term care. Handbook of Clinical Nutrition and Aging. Nutrition and Health.

[3] Amella EJ (1999). Factors influencing the proportion of food consumed by nursing home residents with dementia. J Am Geriatr Soc.

[4] Tamura BK, Masaki KH, Blanchette P (2007). Weight loss in patients with Alzheimer’s disease. J Nutr Elder.

[5] Alzheimer’s Association. Adult Day Centers. [https://www.alz.org/help-support/caregiving/care-options/adult-day-centers] Accessed on 13 Mar 2020.

[6] Matthews KA, Xu W, Gaglioti AH (2019). Racial and ethnic estimates of Alzheimer's disease and related dementias in the United States (2015-2060) in adults aged ≥65 years. Alzheimers Dement.

[7] Sousa L, Sequeira C, Ferré-Grau C, Neves P, Lleixà-Fortuño M (2016). Training programmes for family caregivers of people with dementia living at home: integrative review. J Clin Nurs.

[8] Wolff JL, Mulcahy J, Huang J, Roth DL, Covinsky K, Kasper JD (2018). Family caregivers of older adults, 1999-2015: trends in characteristics, circumstances, and role-related appraisal. Gerontologist.

[9] Mamhidir AG, Karlsson I, Norberg A, Mona K (2007). Weight increase in patients with dementia, and alteration in meal routines and meal environment after integrity promoting care. J Clin Nurs.

[10] Brookdale Foundation Group. The Brookdale Foundation Group – 60 Years. [http://www.brookdalefoundation.net/aboutus.html] Accessed on 13 Mar 2020.

[11] Herke M, Fink A, Langer G (2018). Environmental and behavioural modifications for improving food and fluid intake in people with dementia. Cochrane Database Syst Rev.

[12] Bronfenbrenner U, Evans G (2000). Developmental science in the 21st century: emerging questions, theoretical models, research designs and empirical findings. Soc Dev.

[13] Teerenstra S, Moerbeek M, van Achterberg T, Pelzer BJ, Borm GF (2008). Sample size calculations for 3-level cluster randomized trials. Clin Trials.

[14] Hepburn K, Lewis M, Tornatore J, Sherman CW, Bremer KL (2007). The savvy caregiver program: the demonstrated effectiveness of a transportable dementia caregiver psychoeducation program. J Gerontol Nurs.

[15] McCulloch CE, Searle SR (2001). Generalized, linear, and mixed models.

[16] Hedeker D, Mermelstein RJ, Demirtas H (2007). Analysis of binary outcomes with missing data: missing = smoking, last observation carried forward, and a little multiple imputation. Addiction.

[17] Fitzmaurice GM, Laird NM, Ware JH (2011). Applied Longitudinal Analysis 2nd ed.

[18] Glasgow RE, Vogt TM, Boles SM (1999). Evaluating the public health impact of health promotion interventions: the RE-AIM framework. Am J Public Health.

